# Availability of Medications for Opioid Use Disorder in US Psychiatric Hospitals

**DOI:** 10.1001/jamanetworkopen.2024.44679

**Published:** 2024-11-13

**Authors:** Shawn M. Cohen, Tamara Beetham, David A. Fiellin, Srinivas B. Muvvala

**Affiliations:** 1 Program in Addiction Medicine, Section of General Internal Medicine, Yale School of Medicine, New Haven, Connecticut; 2 Yale School of Public Health, New Haven, Connecticut; 3 Department of Medicine, Yale School of Medicine, New Haven, Connecticut; 4 Department of Emergency Medicine, Yale School of Medicine, New Haven, Connecticut; 5 Department of Psychiatry, Yale School of Medicine, New Haven, Connecticut

## Abstract

This cross-sectional study examines the availability of medications for opioid use disorder in psychiatric hospitals in the US.

## Introduction

In the midst of an unprecedented overdose crisis, medications for opioid use disorder (MOUD), namely methadone, buprenorphine, or naltrexone, remain the most effective treatment for OUD yet remain underutilized.^1^ Expanding availability of MOUD in all health care settings is a critical step in addressing the overdose crisis. Given the high comorbidity of psychiatric illness and OUD, access to MOUD within psychiatric treatment settings could have an outsized potential impact.^2^ We aimed to describe MOUD availability at psychiatric hospitals throughout the US.

## Methods

This is a cross-sectional analysis of facility-level responses to the 2022 National Substance Use and Mental Health Services Survey (N-SUMHSS; 88% response rate).^3^ This study was considered nonhuman participants research by the Yale University institutional review board and did not require informed consent.

The study sample included all facilities in the US that self-reported their facility type as a psychiatric hospital and reported their MOUD availability. MOUD availability was measured as a dichotomous variable indicating whether MOUD (ie, buprenorphine, methadone, and/or naltrexone) is a service psychiatric hospitals report providing. Wilcoxon rank-sum and χ^2^ tests were used to compare MOUD availability by facility characteristics. To assess for potential nonresponse bias on the MOUD provision rate, a Monte Carlo simulation with 1000 imputations was conducted modeling MOUD provision among nonrespondents based on characteristics associated with both MOUD provision and nonresponse. Analyses were conducted from February to September 2024 using Stata version 18.0 MP (StataCorp) with statistical significance defined as 2-tailed with *P* < .05. We followed the STROBE reporting guideline.

## Results

Of 1107 psychiatric hospitals who responded to the survey, information on MOUD provision was available for 1021 (92.23% response rate) (Figure). Facilities were identified as inpatient psychiatric units within a general hospital (558 facilities [54.65%]), freestanding psychiatric hospitals (428 facilities [41.92%]), and state hospitals (35 facilities [3.43%]) (Table).

**Figure.**
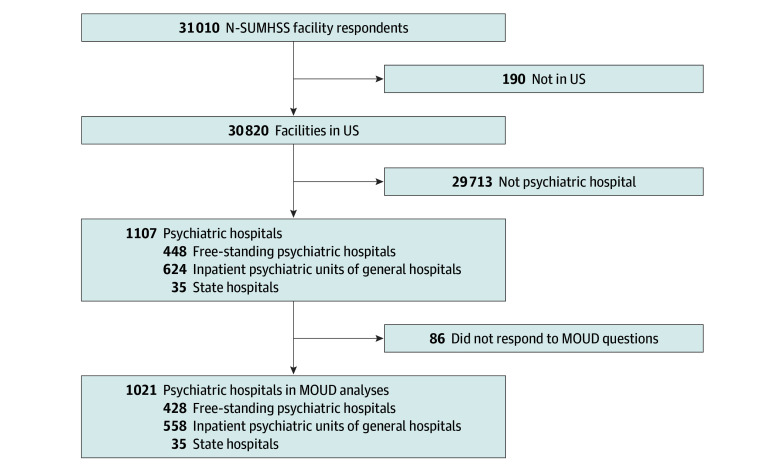
Flow Diagram N-SUMHSS indicates National Substance Use and Mental Health Services Survey; MOUD, medications for opioid use disorder.

**Table.** Psychiatric Hospital Characteristics and MOUD Provision^a^

Characteristic	Hospitals, No. (%)		*P* value^b^
	Total (N = 1021)	Offers MOUD (n =490)	
Facility type			
Freestanding psychiatric hospital	428 (41.92)	200 (46.73)	.49
Inpatient psychiatric unit of a general hospital	558 (54.65)	274 (49.1)	.44
State hospital	35 (3.43)	16 (45.71)	.78
Ownership			
Private nonprofit organization	519 (50.83)	247 (50.2)	.03
Private for-profit organization	290 (28.4)	134 (47.69)	.80
Publicly owned^c^	212 (20.76)	52 (35.86)	.002
Bed capacity, No. (median) [IQR]	937 (30) [18-70]	417 (36) [20-75]	.003
Payers accepted			
Medicare	955 (93.54)	477 (49.95)	<.001
Medicaid	968 (94.81)	470 (48.55)	.13
Other state-financed health insurance	679 (66.50)	340 (50.07)	.06
Private insurance	979 (95.89)	479 (48.93)	.004
Military insurance	700 (68.56)	344 (49.14)	.28
Cash	940 (92.07)	462 (49.15)	.01
Funding accepted			
County or local government	327 (32.03)	148 (47.28)	.68
State agencies^d^	599 (58.67)	295 (49.25)	.34
Federally funded grants^e^	132 (12.93)	64 (48.48)	.90
Veterans Affairs	412 (40.35)	201 (48.79)	.68
Indian Health Services, tribal, or urban	165 (16.16)	78 (47.27)	.84
Private or community foundation	34 (3.33)	21 (61.76)	.10
Age groups accepted			
Children (0-12 y)	214 (21.27)	102 (47.66)	.99
Adolescents (13-17 y)	341 (33.43)	174 (51.03)	.18
Young adults (18-25 y)	872 (85.49)	462 (52.98)	<.001
Adults (26-64 y)	932 (91.37)	480 (51.50)	<.001
Older adults (≥65 y)	913 (89.51)	469 (51.37)	<.001
Substance use services provided			
Integrated MH and SU treatment approach	632 (62.27)	358 (56.65)	<.001
Co-occurring MH and SU group or program	607 (60.10)	344 (56.67)	<.001
Medically managed withdrawal	441 (45.09)	308 (69.84)	<.001
Medications for alcohol use disorder	406 (41.51)	373 (91.87)	<.001
Individual counseling	609 (62.40)	333 (54.68)	<.001
Group counseling	717 (73.39)	403 (56.21)	<.001
12-Step groups	213 (21.82)	146 (68.54)	<.001
Case management	503 (51.54)	271 (53.88)	<.001
Other services provided			
Housing services	87 (8.58)	49 (56.32)	.10
HIV or AIDS group or program	193 (19.15)	108 (56.67)	.01
Traumatic brain injury group or program	129 (12.80)	70 (54.26)	.11
Geographic characteristic			
Medicaid expansion state	737 (72.18)	363 (49.25)	.19
US census region			
Midwest	250 (24.49)	117 (46.80)	.66
Northeast	192 (18.81)	110 (57.29)	.004
South	390 (38.20)	178 (45.64)	.24
West	189 (18.51)	85 (44.97)	.36

Abbreviations: MH, mental health; MOUD, medications for opioid use disorder; SU, substance use.

^a^ Data are from the 2022 National Substance Use and Mental Health Services Survey.

^b^ χ^2^ and Wilcoxon rank-sum tests were used to compare MOUD availability by facility characteristics.

^c^ Publicly owned facilities are those owned by state, local, county, community, tribal, or federal entities.

^d^ State agencies include mental health, welfare or child and family services, state corrections and juvenile justice, education, and/or other state government.

^e^ Federally funded grants include community service block, community mental health block, and other federal grants.

Less than one-half of psychiatric hospitals (490 facilities [47.99%]) reported providing MOUD. Facilities were more likely to provide MOUD if they provided medications for alcohol use disorder (373 facilities [91.87%]; χ^2^_1_ = 568.32; *P* < .001) or medically managed withdrawal services (308 facilities [69.84%]; χ^2^_1_ = 173.94; *P* < .001); of 441 facilities offering medically managed withdrawal, 133 (30.16%) did not provide MOUD. Facilities that provided MOUD had greater median (IQR) bed capacity than the overall sample (30 [18-70] vs 36 [20-75]; *P* = .03), and MOUD provision was positively associated with being located in the Northeast (110 facilities [57.29%]; χ^2^_1_ = 8.19; *P* = .004). Publicly owned psychiatric hospitals were less likely to provide MOUD (52 facilities [35.86%]; χ^2^_1_ = 9.30; *P* = .002). We estimate an adjusted MOUD provision rate of 47.36% (SE = 1.56%) in our robustness check, indicating our reported 47.99% MOUD provision rate was unlikely to be affected by nonresponse bias.

## Discussion

In this cross-sectional study, we found that less than one-half of psychiatric hospital survey respondents provide MOUD. It is additionally concerning that 30% of psychiatric facilities offering medically managed withdrawal did not provide MOUD given the possible harm of this approach including increased risk of overdose.^4^

Consistent with prior research in community outpatient mental health facilities,^5^ our findings suggest that low MOUD availability is a systematic issue in psychiatric care. Stigma against MOUD likely plays a role in these findings. Lack of knowledge about MOUD, its effectiveness, and regulations surrounding its provision also likely contributes.

We suspect our main finding is an overestimate of true availability. Among facilities that reported providing MOUD, the survey did not specify which form of MOUD (methadone, buprenorphine, or naltrexone) was available or the frequency of availability. Availability of more regulated forms of MOUD, namely methadone and buprenorphine, may be lower. Additionally, providing MOUD conditionally or even for a single patient would allow a facility to meet criteria for provision without reflecting true availability.

Limitations include potential upward bias from facilities self-reporting MOUD provision. Social desirability bias may contribute particularly because N-SUMHSS data are publicly available.^6^ This study exposes the deficiency in MOUD access at inpatient psychiatric hospitals and possible avenues to improve MOUD provision particularly to people with comorbid OUD and mental illness.

## Notes

Supplement.
Data Sharing Statement
