# Supplementary material for: Availability of Medications for Opioid Use Disorder in US Psychiatric Hospitals
Source: JAMA Netw Open. 2024 Nov 13;7(11):e2444679. doi: 10.1001/jamanetworkopen.2024.44679 (PMC11561693; doi:10.1001/jamanetworkopen.2024.44679)
Supplement: Supplement. — Data Sharing Statement [file jamanetwopen-e2444679-s001.pdf]

Data Sharing Statement

Cohen. Availability of Medications for Opioid Use Disorder in US Psychiatric Hospitals. *JAMA Netw Open*. Published November 13, 2024. doi:10.1001/jamanetworkopen.2024.44679

Data
Data available: Yes
Data types: Data (not involving human participants)
How to access data: https://www.datafiles.samhsa.gov/dataset/national-substance-use-and-mental-health-services-survey-2022-n-sumhss-2022-ds0001
When available: With publication

Supporting Documents
Document types: None

Additional Information
Who can access the data: Publicly available data
Types of analyses: Publicly available data
Mechanisms of data availability: Publicly available data
